# Editorial Notice

**Published:** 1842-04

**Authors:** 


### THE WESTERN JOURNAL.

Vol. V.—No. IV.

LOUISVILLE, APRIL 1, 1842.

### EDITORIAL NOTICE.

Dr. Drake will spend the ensuing vacation in Cincinnati, and Dr. Yandell, at Murfreesboro', Tennessee. Correspondents of the Journal who reside near those places may direct their communications to either of those gentlemen: all others will please forward them to Dr. Colescott, Louisville.

